# Supplementary material for: Cartilaginous Intrusion of the Atrioventricular Node in a Quarter Horse with a High Burden of Second-Degree AV Block and Collapse: A Case Report
Source: Animals (Basel). 2022 Oct 24;12(21):2915. doi: 10.3390/ani12212915 (PMC9657453; doi:10.3390/ani12212915)
Supplement: Supplementary file 1 [file animals-12-02915-s001.zip › animals-1926554-supplementary.pdf]

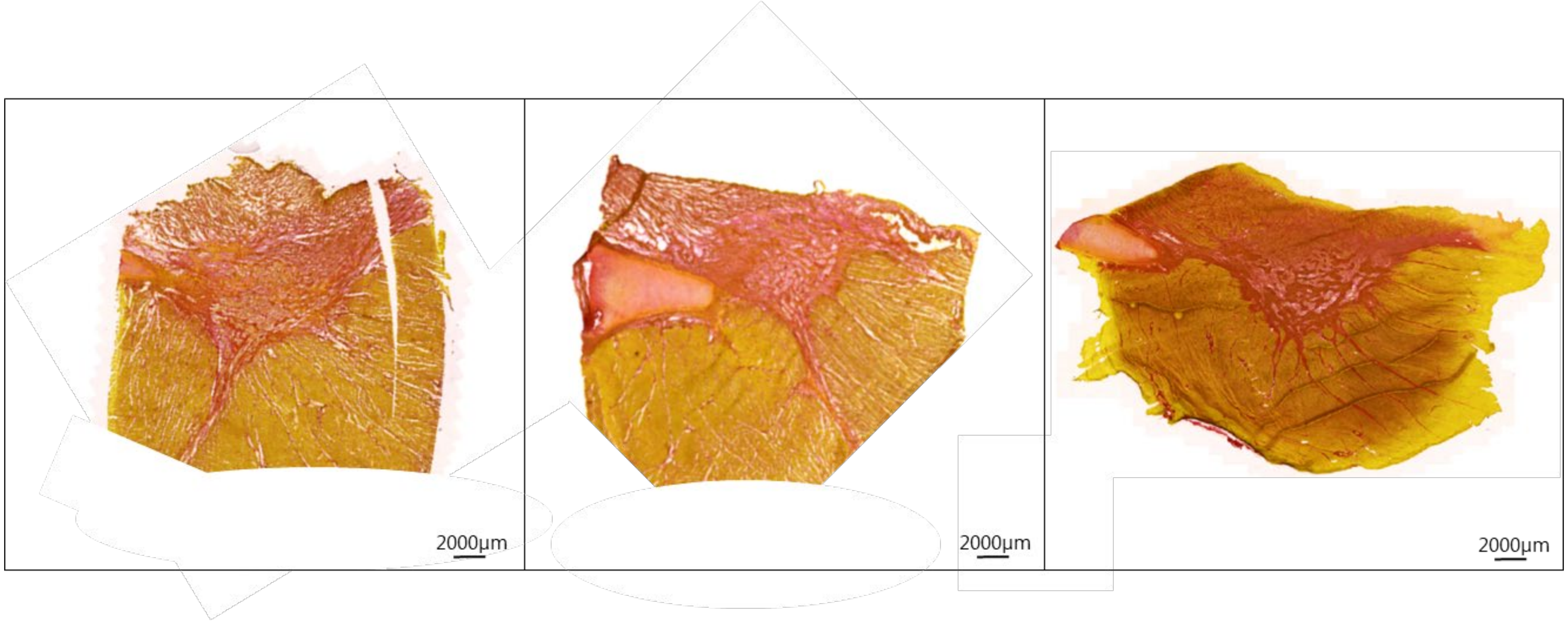

Figure S1. Representative Picro Sirius histology of the His bundle region from three healthy Standardbred racehorses.
